# Genome-Wide Analysis of the Maize LBD Gene Family Reveals a Role for *ZmLBD12* in the Development of Lateral Roots

**DOI:** 10.3390/plants14162600

**Published:** 2025-08-21

**Authors:** Shifeng Wang, Yang Wang, Jianbing Zhong, Wenlin Xu, Qingyou Gong, Lihong Zhai, Gaoke Li, Jun Huang

**Affiliations:** 1 Guangdong Provincial Key Laboratory of Plant Molecular Breeding, College of Agriculture, South China Agricultural University, Guangzhou 510642, China; 20232015043@stu.scau.cn (S.W.); 133249450706@stu.scau.cn (Y.W.); zhongjianbin@stu.scau.cn (J.Z.); 2 Basic School of Medicine, Hubei University of Arts and Science, Xiangyang 441053, China; 2023168181@hbuas.edu.cn; 3 Zhuhai Modern Agriculture Development Center, Zhuhai 519000, China; gongqingyou@126.com; 4 Crops Research Institute, Guangdong Academy of Agricultural Sciences, Guangdong Provincial Key Laboratory of Crop Genetic Improvement, Guangzhou 510640, China

**Keywords:** maize, LBD gene family, *ZmLBD12*, function analysis, lateral root

## Abstract

The growth and yield of the aboveground parts of maize (*Zea mays* L.) are closely associated with development of the root system. LBD (Lateral Organ Boundaries Domain) transcription factors are crucial for the regulation of lateral organ development in plants. However, to date, little information has been uncovered about the LBD gene family in maize. In this research, a genome-wide identification revealed 45 LBD gene members in maize. The subsequent phylogeny, structure, and profiles of expression were analyzed. These genes were found to be dispersed across all 10 maize chromosomes and expressed in diverse tissues, including the roots, leaves, stems, pericarp, and vegetative meristems. Notably, *ZmLBD12* exhibited specific expression in roots. Subsequent over-expression of *ZmLBD12* in *Arabidopsis thaliana* demonstrated its role in lateral root development, identifying it as a candidate gene for further investigation of root development in maize. Our findings provide a systematic analysis of ZmLBD genes and highlight *ZmLBD12* as a potential target gene for developing high-yielding, lodging-resistant maize varieties.

## 1. Introduction

In plants, the root system is an important organ for their growth, development, and adaptation to the environment. The spatial distribution of the root system within the soil, known as root system architecture, directly affects the growth of plants because it has important roles in the absorption of water and nutrients, stabilization of the plants, and environmental adaptation [1]. The number and orientation of lateral roots determine the spatial distribution and area of absorption of root systems in the soil. In maize (*Zea mays* L.), lateral roots expand the absorptive surface area of the root system, thereby significantly enhancing the efficiency of acquisition of nutrients and water [2,3]. Consequently, elucidating the genetic regulatory mechanisms that govern lateral root development is essential for the precision breeding of crop varieties with high-density lateral roots.

The LBD (Lateral Organ Boundaries Domain) gene family, also designated AS2/LOB, is a class of transcription factors (TFs) that is unique to higher plants. It participates in various aspects of plant development. However, the expression of this gene was first discovered at the base of primary lateral organs in *Arabidopsis thaliana* and was designated Lateral Organ Boundaries (*LOB*/*AtASL4*) [4,5]. The LBD proteins harbor a conserved N-terminal LOB domain and a variable C-terminus region. Within the LOB domain, the C-block, which contains four completely conserved cysteine residues and multiple non-conserved amino acid residues, is probably essential to bind DNA [6]. Additionally, the LOB domain harbors a C-terminal leucine-like zip region (LX6LX3LX6L) and an invariant glycine residue (GAS). The C-terminal leucine-like zipper region consists of 19 amino acids and is associated with the dimerization of proteins. The leucine-like region is immediately followed by the variable C-terminus, where the amino acid sequences of the LBD members are highly specific. In addition, the variable C-terminal is linked to nuclear targeting [5,7]. Together, the LBD proteins conclude the LOB domain and variable C-terminal [8]. The LBD gene family is divided into two major categories (Class I and Class II) based on the integrity of the leucine-like zipper motif. The Class I genes possess a complete leucine-like zipper motif, whereas the Class II genes lack this intact structure. Given the close association between the leucine-like zipper region and the dimerization of proteins, Class II LBD proteins cannot form coiled-coil higher-order structures, which may impact the manifestation of their biological functions. Therefore, most of the LBD genes belong to Class I [9].

Currently, the LBD gene family has been comprehensively investigated in model plants, such as *Arabidopsis thaliana* and rice, as well as in many other plants, such as *Populus trichocarpa* and *Salvia miltiorrhiza* [10,11]. Moreover, increasing evidence suggests that these genes are essential for plant development. First, the LBD genes exert significant effects on the development of leaves. For example, *AtAS2* (*AtLBD6*) participates in the formation of symmetric planar leaves by inhibiting the proliferation of cells [10,12], and the *STM* (*SHOOT MERISTEMLESS*) gene regulates the expression of *AtAS2* in the shoot apical meristem (SAM). Concurrently, *AtAS2* interacts with the *AtAS1* and negatively regulates the STM [13]. Additionally, the AS1-AS2 hetero-dimer represses the expression of the *ETT*/*ARF3* and *KANADI2* (*KAN2*), which maintains the determined state of leaf cells [14,15]. Secondly, the LBD genes influence the development of flowers. LBD genes, such as *AtAS2*, *AtAS1*, and *JAGGED* (*JAG*), act synergistically in the floral organs to collectively determine the position of boundary cells [16]. The LBD proteins are also associated with the metabolic processes of plants in addition to their roles in the developmental processes. The over-expression of *AtASL39* (*AtLBD37*), *AtASL40* (*AtLBD38*), and *AtASL41* (*AtLBD39*) in transgenic *A. thaliana* suppresses the expression of genes related to the biosynthesis of anthocyanins, such as *PAP1* and *PAP2*, and the genes involved in nitrogen metabolism. Therefore, these three LBD genes act as negative regulators that affect the biosynthesis of anthocyanins and nitrogen metabolism in plants [17]. In rice (*Oryza sativa*), *OsLBD37* also regulates nitrogen metabolism [11]. Additionally, compared with wild-type (WT) plants, the heterologous over-expression of the *ZmLBD2* gene in *Arabidopsis thaliana* under drought stress significantly reduced the levels of superoxide anion (O_2_^−^) and hydrogen peroxide (H_2_O_2_), while enhancing the activities of catalase (CAT), superoxide dismutase (SOD), and peroxidase (POD). These findings suggest that the gene may enhance drought resistance by reducing ROS accumulation [18]. Third, the LBD genes play crucial roles in the development of roots in both monocots and dicots. For example, the *arf7*/*arf19* double mutant of *A. thaliana* fails to form normal lateral roots, while the over-expression of *AtLBD16* or *AtLBD29* partially rescues the defect of this mutant [19]. *Rtcs*, an LBD gene closely homologous to *AtLBD29*, is expressed in the crown root primordia of maize, and *rtcs* fails to form normal crown roots [20]. Similarly, *OsCrl1*, an ortholog of the maize *rtcs*, is expressed at the initiation site of the crown roots in rice. Moreover, *crl1* does not respond to stimulation by auxin, loses geotropism, and is unable to form normal lateral or adventitious roots [20]. However, WOX11 induces the expression of its downstream gene *LBD16* in adventitious root initial cells. ARF7 and ARF19 also induce the expression of *LBD16* in lateral root initial cells to ensure the development of normal root primordia. Therefore, the early developmental mechanisms of root primordia can serve as the basis to distinguish between lateral and adventitious roots [21,22,23]. Nevertheless, maize lacks a comprehensive genomic characterization of the LBD gene family.

As a vital global source of food, feed, and industrial raw materials, the yield of maize is essential for food security and economic development. Based on the important function of LBD in the development of plants, this study conducted a systematic analysis of the LBD gene family in maize, which laid a foundation for the future exploration of the role of the LBD gene in maize growth and development, as well as stress resistance. Furthermore, we identified *ZmLBD12*, a gene that is specifically expressed in roots, and analyzed its phenotypic characteristics in transgenic *A. thaliana*. These findings may offer a theoretical basis for breeding high-yielding and lodging-resistant maize varieties.

## 2. Results

### 2.1. Identification of the ZmLBD Gene Family

**Figure 1:**
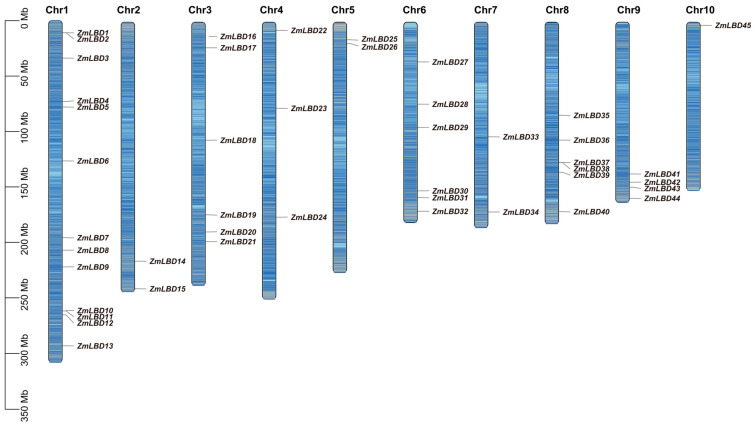
Chromosomal-scale distribution of the ZmLBD genes in maize. The physical positions of the ZmLBD family members are mapped to their respective chromosomes. The gene density on the chromosomes is visualized by the gradient blue bands.

A total of 45 LBD genes in the maize genome were identified based on their domains and the removal of redundant sequences. We renamed these genes as *ZmLBD1* to *ZmLBD45* based on their chromosomal locations [5]. These 45 genes are randomly and unevenly distributed across 10 chromosomes in maize (Figure 1). Chromosome 1 harbors the highest number of LBD genes (13 in total), while chromosome 10 contains only one LBD gene. Additionally, chromosomes 2, 5, and 7 each harbor two LBD genes; chromosome 4 harbors three; chromosomes 3, 6, and 8 each harbor six LBD genes, and the remaining LBD genes are distributed on chromosome 9. This suggests that the LBD genes may be widely present in the genome of the ancestors of maize.

**Figure 2:**
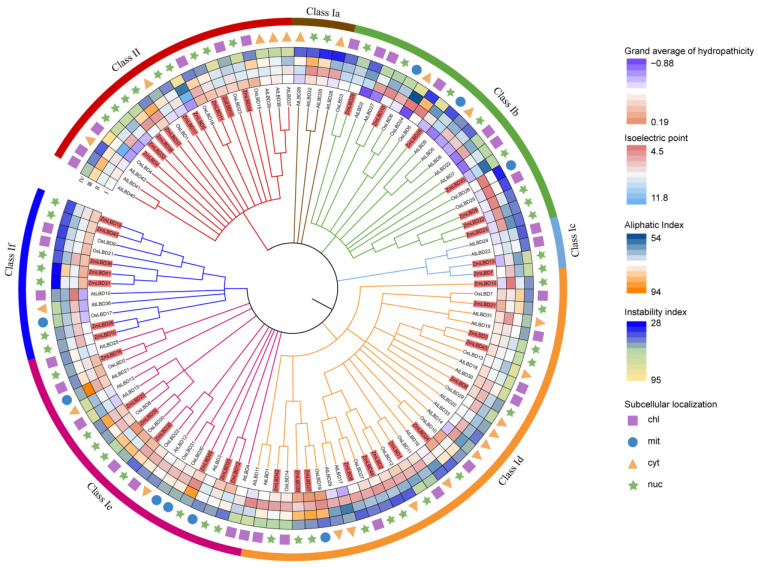
The phylogenetic and functional landscape of the LBD proteins. Maximum likelihood tree constructed with the full-length protein sequences from maize (Zm), rice (Os), and *Arabidopsis* (At). Concentric circles represent (1) physicochemical properties (gradient color scales), (2) predicted localization (symbols defined in key), and (3) subfamily membership (colored arcs). Bootstrap values > 70% shown at the nodes.

The proteins encoded by *ZmLBDs* range from 161 amino acids (ZmLBD33, 17.28 kDa) to 683 amino acids (ZmLBD15, 74.60 kDa). The isoelectric points (pIs) span from 4.52 (ZmLBD15) to 11.74 (ZmLBD5). The instability indices of the LBD gene family range from 28.84 (ZmLBD31) to 89.52 (ZmLBD5), which indicates that there has been substantial functional divergence within this family. This suggests that these genes have distinct transcriptional regulatory roles in specific biological processes. The aliphatic index (AI), another indicator of protein stability, indicates that higher AI values correlate with greater stability. The AI values of the ZmLBDs range from 57.83 (ZmLBD33) to 90.31 (ZmLBD45) (Figure 2 and Table 1). Notably, the prediction of subcellular localization indicates that all maize LBD proteins are located in the nucleus, while LBD proteins in *Arabidopsis thaliana* and rice are located in the cytoplasm, nucleus, mitochondria, and chloroplast.

### 2.2. Phylogenetic Relationship of the LBD Proteins Among Maize, Rice, and Arabidopsis thaliana

We conducted a phylogenetic analysis using the LBD protein sequences from maize, rice, and *A. thaliana* to elucidate the evolutionary relationships of the LBD gene family. All of the LBD proteins were found to be classified into two major categories (Group I and Group II). Additional analysis revealed that Group I could be subdivided into six subgroups. The largest subgroup, Id, contained 13 ZmLBD members. Notably, the ZmLBD genes were only absent from Group Ia, which is consistent with the observation of previous studies [4]. Group II contained only 20 LBD proteins, including the following nine maize LBDs: ZmLBD4, ZmLBD5, ZmLBD11, ZmLBD14, ZmLBD17, ZmLBD32, ZmLBD34, ZmLBD37, and ZmLBD38 (Figure 2). The remaining LBD proteins were classified into Group I. The phylogenetic tree revealed a close relationship between maize and rice. This further confirmed the evolutionary connections among these two species.

### 2.3. Gene Structure and Protein Motifs of the ZmLBDs

**Figure 3:**
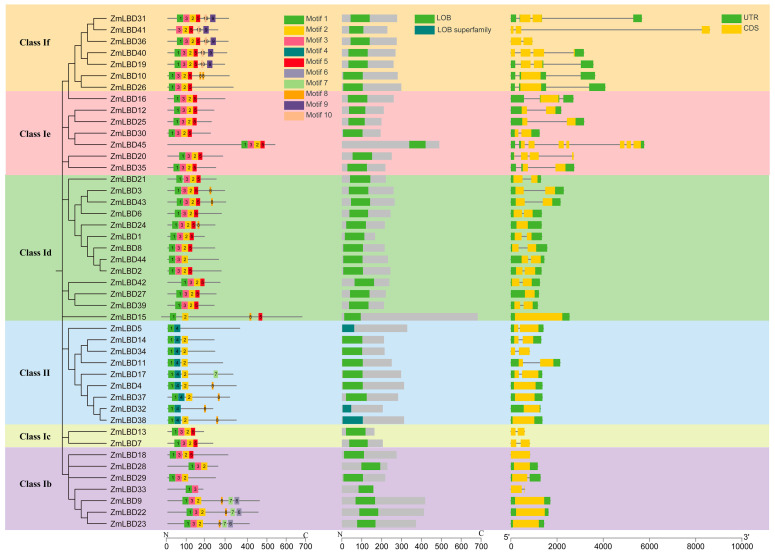
Structural characterization of the ZmLBD genes in maize. The figure combines the following three analyses: (1) motif composition identified using MEME Suite (left panel); (2) organization of conserved domains (middle panel); (3) gene structure with coding sequences (CDSs) and untranslated regions (UTRs) in yellow (right panel). Colored background areas denote phylogenetic subfamilies.

The potential functions and regulatory mechanisms of the ZmLBDs were revealed by conducting a structural analysis using TBtools (v. 2.142). The MEME Suite 5.5.7 online software revealed a total of 10 motifs, and all of the ZmLBDs contained an LOB domain (Figure 3 and Table S1). Furthermore, the relative positions of these motifs were similar. Currently, the ZmLBDs contain three or more conserved motifs. All of the ZmLBDs except ZmLBD32 contain Motifs 1 and 2, which represent the core conserved parts of the LOB domain. Interestingly, certain motifs were only found in specific subfamilies. For example, Motif 4 was only detected in Subfamily II, while Motif 3 was only present in Subfamily I (Figure 3). We also examined the gene structure by analyzing the intron and exon distribution of the ZmLBDs (Figure 3). Notably, the exon–intron architectures of the LBD genes within the same phylogenetic branch were typically similar. However, the intron numbers in the maize LBD genes ranged from one to seven. A total of 24 genes contained two introns, 16 genes contained one intron, 2 genes contained three introns, and 1 gene (*ZmLBD45*) contained seven introns. This variability highlights the diversity of maize LBD genes.

### 2.4. cis-Regulatory Elements in the Promoters of ZmLBD Genes

**Figure 4:**
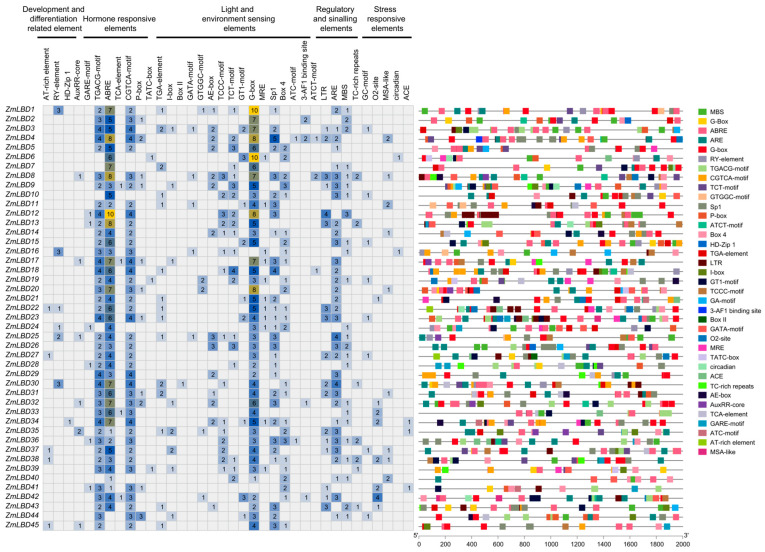
*cis*-regulatory element profiling in the ZmLBD promoters. The number of five types of *cis*-regulatory elements in the promoter sequences of ZmLBD genes is shown. The 2 kb promoter region upstream of each ZmLBD gene is scaled uniformly (bottom axis).

We predicted the *cis*-acting elements in the 2000 bp promoter sequence upstream of ZmLBD genes to discover the potential roles of these genes within the regulatory networks. A total of 36 key *cis*-regulatory elements were identified. They are associated with plant growth, defense, hormone signaling, light response, and stress responses (Figure 4). Except for *ZmLBD44* and *ZmLBD45*, most of the ZmLBD gene promoters contained the ABA-responsive element ABRE, and 11 ZmLBD genes (24.4%) harbored more than seven ABREs (Figure 4). The promoter region of *ZmLBD4* contains eight ABREs, which can participate in regulating maize growth and drought response by affecting the synthesis of ABA [24]. The promoters of 40 ZmLBD genes (88.9%) contained the jasmonate-responsive element CGTCA-motif. Moreover, except for *ZmLBD16* and *ZmLBD41*, all of the other ZmLBD gene promoters contained light-responsive elements. In fact, nine ZmLBD genes (20%) had more than seven G-box elements (Figure 4).

### 2.5. Synteny and Collinearity of the ZmLBD Proteins

**Figure 5:**
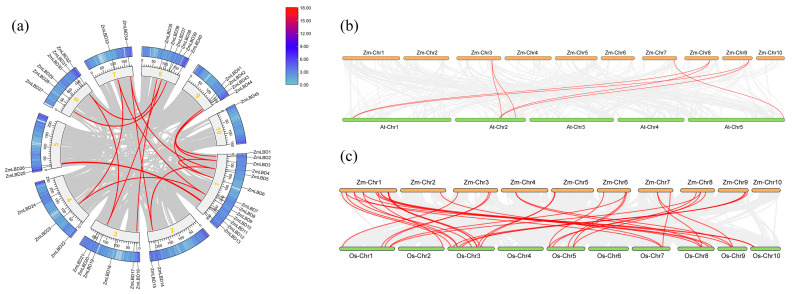
Comparative synteny analysis of the LBD gene families. (**a**) Intraspecies synteny in maize. Circos plot, collinear LBD gene pairs (red lines) across 10 chromosomes (inner ring, numbered in yellow). Peripheral bar charts, gene density (gradient shading: dark = high density). (**b**) Maize–*Arabidopsis* (dicot) synteny: the top represents 10 chromosomes of maize and the bottom represents 5 chromosomes of *Arabidopsis thaliana*; red lines connect orthologous LBD gene pairs. (**c**) Maize–rice (monocot) synteny: the top represents the ten chromosomes of corn and the bottom represents the ten chromosomes of rice; orthologous pairs linked by red lines.

A syntenic analysis was conducted in maize and across different species to reveal the functional conservation and evolutionary history of LBDs. A total of 18 syntenic gene pairs within maize were identified. In particular, they included the following: *ZmLBD2*/*ZmLBD44*, *ZmLBD3*/*ZmLBD6*, *ZmLBD3*/*ZmLBD43*, *ZmLBD5*/*ZmLBD14*, *ZmLBD5*/*ZmLBD34*, *ZmLBD6*/*ZmLBD43*, *ZmLBD9*/*ZmLBD23*, *ZmLBD9*/*ZmLBD33*, *ZmLBD10*/*ZmLBD26*, *ZmLBD12*/*ZmLBD25*, *ZmLBD14*/*ZmLBD34*, *ZmLBD23*/*ZmLBD33*, *ZmLBD27*/*ZmLBD39*, *ZmLBD19*/*ZmLBD31*, *ZmLBD31*/*ZmLBD36*, *ZmLBD16*/*ZmLBD35*, *ZmLBD19*/*ZmLBD36*, and *ZmLBD32*/*ZmLBD37* (Figure 5a). Notably, the *ZmLBD2* gene and *Zm00001eb191170* formed a homologous pair, but *Zm00001eb191170* has not yet been confirmed as an LBD family member. This suggests that the loss of potential gene function occurred, or that there was conserved domain deletion during gene duplication or genome rearrangement. Thus, we evaluated the selection pressures on these syntenic gene pairs, including synonymous mutations (Ks), nonsynonymous mutations (Ka), and their ratio (Ka/Ks). The Ks values ranged from 0.08 to 1.17. The Ks value of the *ZmLBD5*/*ZmLBD34* gene pair was the highest, indicating that the gene pair had been differentiated as early as in the early stage of evolution. All of the duplicate gene pairs had Ka/Ks values < 1.0, which ranged from 0.23 to 0.92 (Table S2). The Ka/Ks ratio values below 1 indicate purifying selection, reflecting evolutionary pressure to conserve gene function. These values indicated that monocot corn and dicot *Arabidopsis thaliana* shared 7 pairs of orthologous genes (Figure 5b), while the same monocot species (corn and rice) exhibited 35 pairs of orthologous genes (Figure 5c). This demonstrates that, the closer the phylogenetic relationship between species, the higher the degree of LBD gene homology. This further illustrates the evolutionary relationship and genomic conservation between the two species.

### 2.6. Analysis of the Pattern of Expression of the ZmLBD Genes

**Figure 6:**
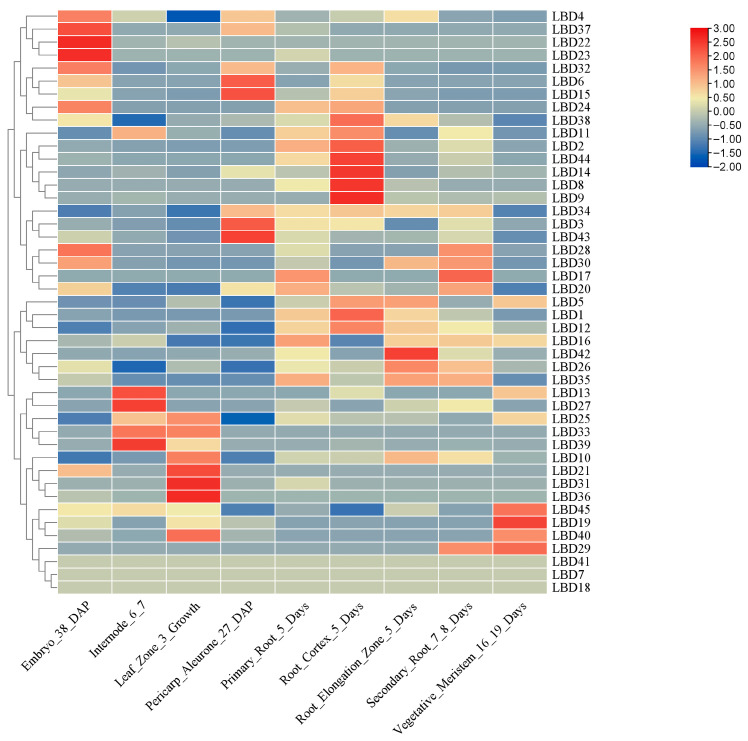
Patterns of expression of the ZmLBDs in nine different tissues of maize.

The expression architectures of the *ZmLBD*s were deciphered across nine tissues by analyzing the RNA-seq data of maize organs at different developmental stages, including embryos at 38 days post-fertilization, internodes at 6–7 days, leaf regions at 3 days of growth, pericarp at 27 days post-fertilization, primary roots at 5 days, root cortex at 5 days, root elongation zones at 5 days, secondary roots at 7–8 days, and vegetative meristems at 16–19 days (Figure 6). Expression profiling showed that the three members *ZmLBD41*, *ZmLBD7*, and *ZmLBD18* were not detected, while the remaining members were expressed in these tissues. *ZmLBD10*, *ZmLBD21*, *ZmLBD25*, *ZmLBD31*, *ZmLBD33*, *ZmLBD36*, and *ZmLBD40* were highly expressed in the leaves, which suggests their potential involvement in photosynthesis and the utilization of nutrients. Moreover, *ZmLBD13*, *ZmLBD27*, *ZmLBD33*, and *ZmLBD39* were highly expressed in the internodes, which indicated important roles in plant growth and development. *ZmLBD34*, *ZmLBD5*, *ZmLBD1*, and *ZmLBD12* were expressed at significantly higher levels in the root tissues compared to other homologous genes (Figure 6). *ZmLBD12* was exclusively highly expressed in the roots. Based on these findings, *ZmLBD12* was selected for further functional characterization.

### 2.7. Transgenic ZmLBD12 Affects the Growth of Lateral Roots in Arabidopsis thaliana

**Figure 7:**
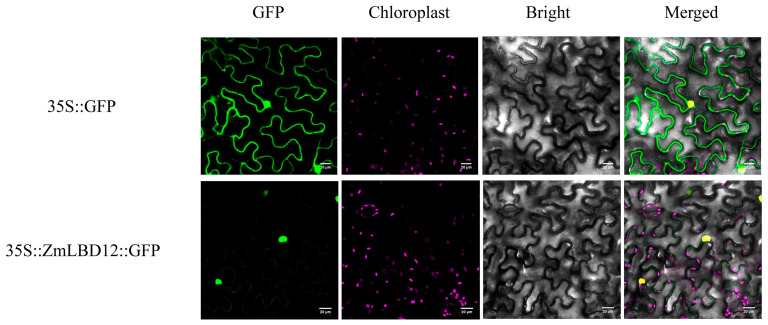
The subcellular localization of 35S::ZmLBD12-GFP in *Nicotiana benthamiana*.

**Figure 8:**
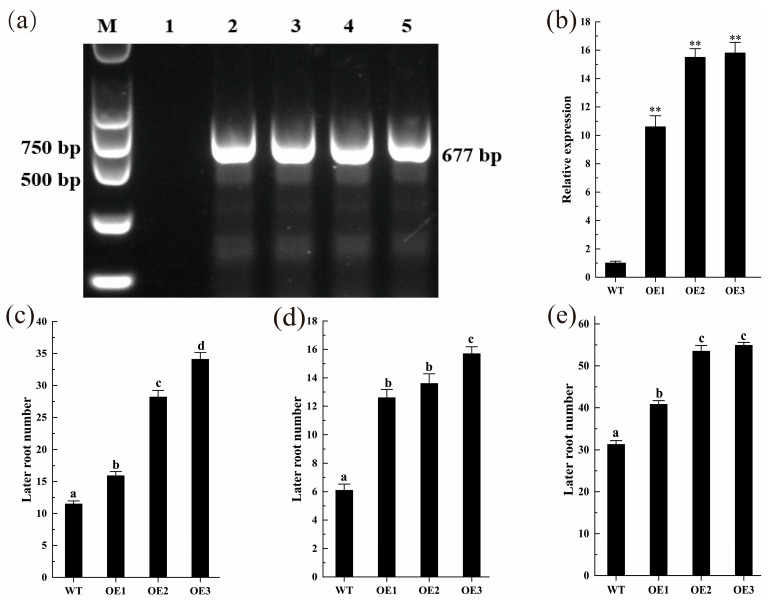
Phenotypic analysis of *Arabidopsis thaliana* over-expressing *ZmLBD12*. (**a**) PCR identification. M: DNA maker; 1: untransformed negative plants; 2: recombinant plasmid-positive control; 3–5: transgenic-positive plants. (**b**) Expression identification of *ZmLBD12.* ** *p* < 0.01. (**c**–**e**). The number of lateral roots of *ZmLBD12*-transformed *A. thaliana* at 6, 12, 18, and 24 days old was counted. The letters above the columns represent significant differences (*p* < 0.05).

**Figure 9:**
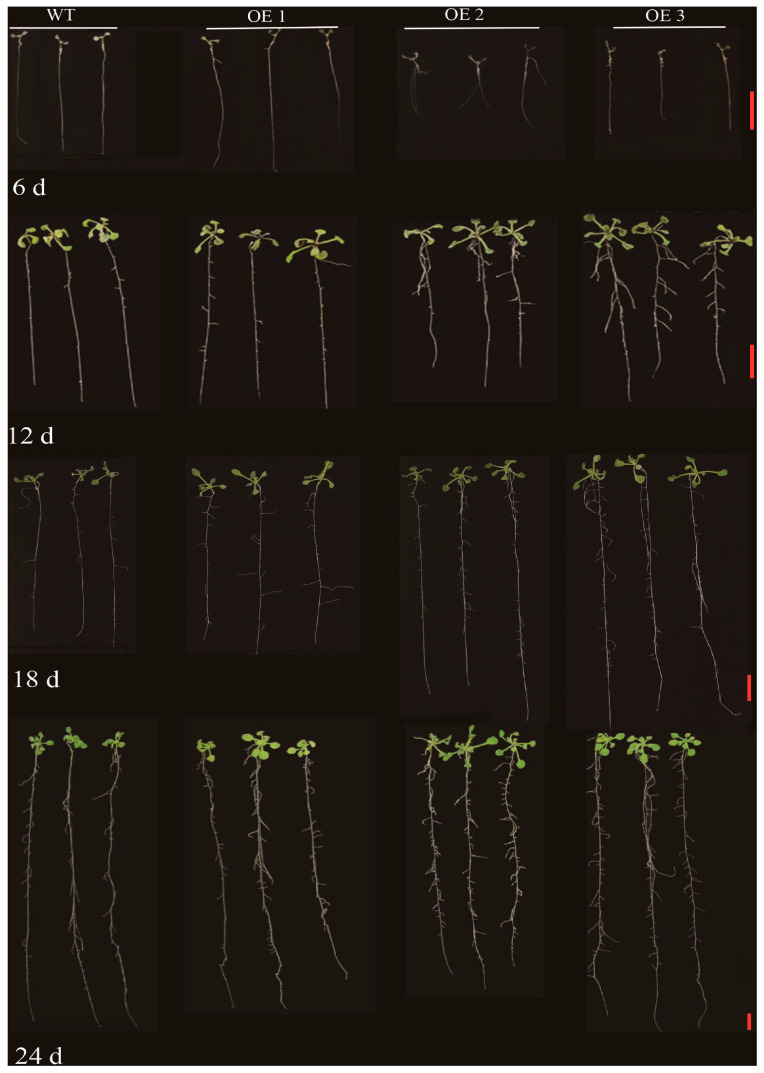
Phenotype of *ZmLBD12*-transformed *Arabidopsis thaliana* at 6, 12, 18, and 24 days old. The red lines in the picture represent a scale of 1 cm.

Previous studies have shown that the LOB domain has a nuclear localization signal [25]. The localization of 35S::ZmLBD12-GFP in tobacco (*Nicotiana benthamiana*) was detected to verify the subcellular localization of ZmLBD12. The ZmLBD12-GFP fusion protein was localized in the nucleus, which was consistent with the nuclear localization of the control 35S::GFP (Figure 7). Three over-expression lines (OE1, OE2, and OE3) were generated by heterologous expression in the WT *A. thaliana* to investigate the effect of *ZmLBD12* on root growth; after being verified by agarose gel electrophoresis, their gene expression levels were verified (Figure 8a,b). Lateral roots emerged in the over-expression lines at 6 days post-germination, whereas no lateral roots were observed in the WT seedlings. The number of lateral roots in all the over-expression lines was significantly higher at 12 days than in the WT. In particular, OE3 had the highest average lateral root number (15.7), followed by OE1 (12.6) and OE2 (13.6), compared to only 6.1 in the WT (Figure 8c and Figure 9). At 18 days, the lateral root counts in the over-expression lines remained significantly higher. The WT, OE1, OE2, and OE3 averaged 11.5, 15.9, 28.2, and 34.1, respectively (Figure 8d and Figure 9). The over-expression lines continued to exhibit higher lateral root numbers by 24 days (31.3 in WT vs. 40.8, 53.5, and 54.9 in OE1, OE2, and OE3, respectively) (Figure 8e and Figure 9).

In summary, the pattern of expression of the maize *ZmLBD* genes is closely associated with the growth, development, and stress responses of the plants, and *ZmLBD12* was shown to specifically regulate the development of lateral roots in *A. thaliana*. However, the underlying molecular mechanisms merit further study to provide theoretical insights to improve the stress resistance and yield of maize, as well as to breed high-yielding varieties of maize.

## 3. Discussion

The LBD family is a group of TFs that are specific to plants. They originated from early terrestrial algae and are widely distributed in plants [26,27]. These factors are pivotal at modulating the growth, development, and initiation of lateral organs, such as leaves, flowers, and lateral roots. Studies on the LBD gene family have been reported in various plants, including *A. thaliana* [5], potato (*Solanum tuberosum*) [28], and tomato (*Solanum lycopersicum*) [29]. However, there is little information on the LBD family in maize. As a crucial part of the maize root system, the development and distribution of lateral roots (LRs) significantly impact the absorption of nutrients by the plants and their growth. Thus, the LRs are vital for the final yield and quality of this crop. Studying the role of LBD genes in lateral root development provides a new perspective to breed high-quality varieties of maize with optimized root systems.

In this study, 45 *ZmLBD* genes were identified in maize. These genes were classified into two major categories (Class I and Class II) based on the integrity of their leucine-like zippers. Class I is further subdivided into five subclasses. A total of 37 genes, accounting for 82% of the total, are members of Class I (Figure 1). Previous studies have shown that most of the LBD genes fall into Class I. For example, 43 LBD genes were identified in the dicot *A. thaliana*, and 37 (86%) were members of Class I [5]. In the dicot rapeseed (*Brassica napus*), 62 LBD genes were identified. A total of 51 are members of Class I and account for 82% of the family [30]. This suggests that this phenomenon may be related to the evolutionary history, functional differentiation, and species-specific adaptation of these gene families [27]. An analysis of the gene structure reveals structural variations among members within the same subcategory. For example, the number of introns in the *ZmLBD* genes within the If subcategory ranges from one to three (Figure 3). We hypothesized that members of the If subcategory may have undergone gene segment splicing or insertion during evolution [31,32]. However, the presence of similar conserved sequences and gene structures within the LBD subclasses suggests that genes within the same subclass may share similar biological functions [33]. Notably, the Ia subclass contains *A. thaliana* LBD genes but lacks orthologs in maize and rice (Figure 2). This suggests that the Ia subclass might be a subfamily that is specific to dicots. A comparison of the LOB-conserved domains revealed that all of the Class I genes except for *ZmLBD41*, *ZmLBD33*, and *ZmLBD15* contain a complete leucine-like zipper motif. This indicates that motifs in the LBD family have been widely conserved during evolution [6].

The *cis*-regulatory elements in the gene promoter region are strongly related to its pattern of expression [34]. We identified 36 distinct *cis*-regulatory elements in the ZmLBD promoter regions, including TGACG-motif, ABRE, CGTCA-motif, ARE, and G-box, which are associated with plant growth, hormone signaling, and stress responses. Among these, elements responsive to auxin, methyl jasmonate, ABA, light, and low temperature are the most common in the promoter regions of LBD gene families across other species [35,36]. Transcriptional regulation that responds to stress represents a fundamental adaptive strategy in plants, and the dynamic modulation of gene expression networks confers phenotypic plasticity under abiotic/biotic constraints [37]. Previous studies have also shown that four *AtLBD* genes (*AtLBD16*, *AtLBD17*, *AtLBD18*, and *AtLBD29*) in *A. thaliana* are regulated by auxin to control the formation of calli and lateral roots [38]. The TGA gene family regulates the resistance of plants and the development of their growth by binding to the TGACG-motif region in the promoters of target genes to activate or repress the transcription of downstream target genes. For example, TGA1 can bind to the promoters of the nitrate transporter genes *NRT2.1* and *NRT2.2* to regulate the responses of plants to nitrogen, which can then affect the growth of primary and lateral roots [39]. The Auxin Response Factor (ARF) family proteins recognize and bind to the Auxin Response Element (ARE) via their DNA-binding domains [40]. AtARF7/AtARF19 bind to the ARE in *A. thaliana* and activate the expression of genes, such as *AtLBD16*, and promote the differentiation of pericycle cells into lateral root primordia [41]. ABA inhibits the activation of lateral root primordium meristems. ABI5, a key TF in the ABA signaling pathway, binds to the promoter regions of genes that contain the ABRE (Abscisic Acid Response Element) to regulate downstream gene expression, thereby influencing cell division and differentiation in lateral root meristems. For example, the *abi8* mutant is insensitive to ABA owing to the lack of an ABI5-encoded bZIP TF binding protein in its lateral root apical meristem cells. This protein plays a crucial role in the initiation and maintenance of lateral root apical meristems, thus indicating that ABRE-mediated ABA signaling is essential to regulate the activity of lateral root meristems [42]. In summary, maize LBD genes may play important roles in the development of plant roots, regulation of hormones, and abiotic stress responses. However, the patterns of expression of different LBD genes merit further verification through molecular biology experiments.

Gene duplication acts as a key driver of evolutionary innovation and generates genetic novelty that enables functional diversification and adaptive evolution [27]. There are two primary evolutionary patterns of gene duplication. They include segmental duplication and tandem duplication [43]. Segmental duplication events are the primary mechanism that drive the expansion of gene families. These events account for more than 92% of all the replication events [44], whereas tandem duplications are relatively rare. These patterns of duplication contribute to functional redundancy and evolutionary diversification among the members of gene families [45]. The maize genome has 18 pairs of repeated LBD genes, including 17 fragment repeat pairs and 1 tandem repeat pair. This is similar to the three tandem repeat events reported in 131 LBD genes in upland cotton (*Gossypium hirsutum*) identified in previous studies [46]. Fragment duplication has dominated the expansion of the maize LBD gene family. Each pair of duplicated genes belongs to the same subgroup, indicating that these chromosomal fragments may have experienced duplication events during evolution but did not fully differentiate, which may lead to functional redundancy. While tandem duplication contributes to local gene expansion, segmental duplication preserves functional modules, promotes adaptive differentiation, and synergistically interacts with other mechanisms to shape plant genome diversity [47]. A comparative analysis of the maize genome with those of two other sequenced plants showed that maize and monocot rice showed significant collinearity in the LBD family members, while there was only a small amount of collinearity in the LBD family members of the dicotyledonous plant *A. thaliana* (Figure 5a). This is consistent with the evolutionary relationship between dicots and monocots.

To comprehensively decipher the biological function of the *ZmLBD12* gene, this study utilized genetic transformation techniques to successfully introduce the *ZmLBD12* gene into the WT *A. thaliana*. Rigorous screening obtained three transgenic lines with high-level gene expression. A phenotypic analysis demonstrated that, when compared with the WT plants, the transgenic *A. thaliana* lines exhibited a significantly increased number of lateral roots at 12, 18, and 24 days post-growth (Figure 8). This finding directly validates that the *ZmLBD12* gene plays a regulatory role in the growth of lateral roots in *A. thaliana*.

Previous molecular genetics and physiological studies on model plants have established that the initiation and development of lateral root primordia depend highly on the auxin signaling pathway [48]. Notably, analysis of the *cis*-acting elements within the promoter region of the *ZmLBD12* gene revealed an enrichment of ABA-responsive ABRE elements. Existing research indicates that the ABI3-ERF1 module plays a pivotal role in mediating crosstalk between ABA and auxin signals during lateral root initiation [49]. While these observations suggest a potential link between *ZmLBD12* and hormonal pathways, the precise role of *ZmLBD12* in mediating ABA–auxin interactions during lateral root development remains unclear. Future studies are needed to determine whether *ZmLBD12* functionally integrates these signals in plants.

## 4. Materials and Methods

### 4.1. Identification of the LBD Family Genes and Sequence Analysis

The genomic and protein sequence data for maize were obtained from the MaizeGDB database (https://maizegdb.org/, accessed on 11 February 2025), and Zm-B73-REFERENCE-NAM-5.0 was selected as the reference sequence version. The contoured Hidden Markov Model for the LOB structural domain (PF03195) (HMM) was retrieved from the Pfam 37.2 database [50]. HMMER 3.0 and TBtools 2.142 software were used to identify the ZmLBDs, and the online SMART 9 (http://smart.embl-heidelberg.de/, accessed on 15 March 2025) and Conserved Structural Domain Database (CDD 3.20, https://www.ncbi.nlm.nih.gov/Structure/bwrpsb/bwrpsb.cgi, accessed on 15 March 2025) were used for further validation [51,52]. We named them *ZmLBD1*-*ZmLBD45* sequentially according to their position on the chromosomes. Localization on the chromosomes was visualized using TBtools v. 2.142 software, and the physicochemical properties of ZmLBD proteins, including the relative molecular weight (MW) and theoretical isoelectric point (pI), were determined by EXPASY (https://web.expasy.org/compute_pi/, accessed on March 16 2025) [53]. The subcellular localization was predicted using the Cell-PLoc2 website (http://www.csbio.sjtu.edu.cn/, accessed on 16 March 2025) [54]. The construction of the phylogenetic tree involved two steps. First, the MEGA7 [55] software was used for a multiple sequence alignment of all the LBD protein sequences, with global pair settings and a maximum of 1000 iterations. Subsequently, the Treebest v. 1.9.2 software was utilized to generate the final file via the Neighbor-Joining (NJ) method and the Jones–Taylor–Thornton (JTT) substitution model.

### 4.2. Analysis of Gene Structural and cis-Regulatory Elements in the ZmLBD Gene Family

The MEME Suite website (https://meme-suite.org/meme/, accessed on 18 December 2024) was used to analyze conserved motifs in the LBD proteins. The motifs and gene structures were visualized using TBtools software [56]. In addition, the PlantCARE database [57] (https://bioinformatics.psb.ugent.be/webtools/plantcare/html/, accessed on 19 December 2025) was utilized to predict the *cis*-regulatory elements.

### 4.3. Gene Duplication Events of the LBDs Among Different Plants

The TBtools software [56] with the One Step McScanX-Super Fast module (with the e-value set to 1 × 10^−5^ and the number of BLAST hits set to 10) was used for syntenic characterization. The Dual Synteny Plot module in TBtools was then used for further visualization.

### 4.4. Analysis of the Transcriptome Data for the ZmLBDs

Expression patterns of maize LBD genes across tissues (the internode, primary root, secondary root, embryo, leaf, root elongation zone, root cortex, pericarp/aleurone, and vegetative meristem) were obtained from MaizeGDB qTeller (https://qteller.maizegdb.org/genes_by_name_B73v5.php/, accessed on 18 March 2025) and visualized by TBtools. The data set is numbered (Walley Atlas 2016 [Briggs Lab]) [58].

### 4.5. Determination of the Subcellular Localization of ZmLBD12

The recombinant 35S::ZmLBD12::GFP fusion vector and the control 35S::GFP were transformed into *Agrobacterium tumefaciens* GV3101. The GFP signal was observed by a laser confocal microscope (OLYMPUS, Tokyo, Japan) after 72 h of low-light conditions. The primer information used in this study is listed in Tables S3 and S4.

### 4.6. ZmLBD12 Gene Cloning and Real-Time Fluorescent Quantitative PCR

Total RNA from the roots of the 7-day self-pollinated maize inbred line B73 was extracted using the Novozymes RNA extraction kit and reverse-transcribed with the Novozymes reverse transcription kit. The full-length *ZmLBD12* sequence was amplified using PCR (PTC-100, MJ Research, Waltham, MA, USA), with cDNA as the template, using a reaction volume of 25 μL. The reaction mixture contained 1 μL of Phanta Max Super-Fidelity DNA Polymerase, 25 μL of 2× Phanta Max Buffer, 1 μL of dNTP Mix (10 mmol/L), 1 μL of forward primer LBD12 (OE)-F (10 μmol/L), 1 μL of reverse primer LBD12 (OE)-R (10 μmol/L) (Table S3), 2 μL of cDNA template, and 19 μL of ddH_2_O. The PCR program was as follows: 95 °C for 3 min, 95 °C for 15 s, 71 °C for 15 s, 72 °C for 20 s, and 72 °C for 5 min for 35 cycles. The amplified products were recovered using a Takara Mini BEST Agarose Gel DNA Extraction Kit v. 4.0 (Takara Bio Inc., Kusatsu, Japan). The homologous recombinase was used to ligate the *ZmLBD12* gene into the VC019 Cloning Vector, and it was then transformed into the *E. coli* receptor DH5α. The single clones were picked and subjected to colony PCR, and those that met the requirements were sent to Guangzhou Kinko Biological Company (Guangzhou, China) for sequencing. The results of the sequencing were analyzed by a Snap Gene comparison.

Fluorescence quantitative PCR (qPCR) primers were designed based on the CDS sequence of *ZmLBD12*. Using cDNA as the template, real-time qPCR was performed with the 2× ChamQ SYBR qPCR Master Mix, and the target gene expression levels were detected using a Bio-Rad CFX96 Real-Time PCR Detection System. The reaction volume was 20 μL. The reaction mixture contained 10 μL of 2× ChamQ SYBR qPCR Master Mix, 1 μL of forward primer (10 μmol/L), 1 μL of reverse primer (10 μmol/L) [18] (Table S4), 2.5 μL of cDNA template, and 6.7 μL of ddH_2_O. The amplification program was set as follows: 95 °C for 3 min, 95 °C for 30 s, followed by 60 °C for 30 s for 39 cycles. This experiment included 3 biological replicates, with each reaction system undergoing 3 technical replicates. Experimental data were processed using the 2^−△△Ct^ method [59] to calculate relative gene expression levels. Significance levels were analyzed using SPSS 23.0 software, and graphs were generated with Origin 2023b software.

### 4.7. Genetic Transformation of ZmLBD12 into Arabidopsis thaliana

The recombinant plasmid pBWA(V)KS-*ZmLBD12* was transformed into *Agrobacterium tumefaciens* GV3101, which was subsequently cultured in LB liquid medium (1 mL) containing the corresponding antibiotics at 28 °C, and centrifuged at 200 rpm for 12 h. The proportion was then expanded to 100 mL, and the incubation was continued to an OD_600_ of approximately 0.8, and the bacterial solution was centrifuged at 3500 rpm for 10 min. The supernatant was poured off, and the strain was resuspended using buffer solution (5% sucrose, 1/2 MS, and 0.03% Silwet L-77) to adjust the OD_600_ to approximately 0.8. *Arabidopsis* Col-0 was used as the background. The plants were watered the day before the treatment to ensure that they were humid. On the treatment day, the flowers that had already opened were cut off, and only the flower buds that just showed white were left on the plant. The flower heads were soaked in the bacterial solution for 20–30 s. The treated plants were placed in a cardboard box prepared in advance, wrapped with black cloth, and put back to the normal conditions of cultivation after 24 h. The seeds of the *Arabidopsis* transgenic T0 generation were harvested after the fruit pods of the plants had ripened.

The pure T2 generation seeds obtained after screening were spotted in 1/2 MS medium and subsequently cultured in the dark at 4 °C for 72 h. After that, they were transferred to an artificial climatic chamber and cultured in the light at 22 °C for 16 h and in the dark at 18 °C for 8 h. The seeds were then incubated in the dark at 4 °C for 72 h and then transferred to an artificial climatic chamber for 8 h. The number of lateral roots in *Arabidopsis thaliana* was observed on days 6, 12, 18, and 24, respectively.

## 5. Conclusions

This study provides the first comprehensive genomic characterization of the LBD transcription factor family in maize, elucidating their evolutionary dynamics and developmental regulation patterns. A total of 45 ZmLBD genes were identified, and systematic phylogenetics, gene structures, and synteny were analyzed. These genes were classified into Classes I and II. Expression profiling in maize and heterologous functional assays in *Arabidopsis* suggest that *ZmLBD12* may contribute to lateral root development. However, its specific molecular mechanisms require further investigation. To gain deeper insights into the function of *ZmLBD12*, future studies should generate transgenic maize lines to determine whether *ZmLBD12* exhibits similar functions in maize. Such research will help to elucidate its molecular regulatory pathways and provide a possible theoretical basis to breed high-yielding varieties of maize that are resistant to lodging.

## Figures and Tables

**Table 1:** Information of the ZmLBD gene family in maize.

Gene Name	Gene ID (Zm-B73-REFERENCE-NAM-5.0)	Amino Acid Length	Molecular Weight	Isoelectric Point	Instability Index	Aliphatic Index	Grand Average of Hydropathy (GRAVY)
*ZmLBD1*	*Zm00001eb003910*	167	17,442.63	6.27	66.36	63.95	−0.131
*ZmLBD* *2*	*Zm00001eb003920*	244	25,470.18	5.66	42.7	67.91	−0.165
*ZmLBD* *3*	*Zm00001eb010570*	260	26,838.36	7.76	67.18	71	−0.269
*ZmLBD* *4*	*Zm00001eb019930*	313	33,961.79	6.14	71.23	71.5	−0.637
*ZmLBD* *5*	*Zm00001eb020740*	329	36,067.36	11.74	89.52	58.81	−0.661
*ZmLBD* *6*	*Zm00001eb026870*	245	25,337.54	6.36	54.46	69.63	−0.149
*ZmLBD* *7*	*Zm00001eb035850*	206	21,561.19	6.64	72.17	66.94	−0.338
*ZmLBD* *8*	*Zm00001eb038820*	215	23,764.66	5.7	43.93	65.91	−0.305
*ZmLBD* *9*	*Zm00001eb042180*	419	45,005.73	4.84	47.25	71.36	−0.399
*ZmLBD* *10*	*Zm00001eb051480*	281	30,137.82	6.41	55.28	72.28	−0.342
*ZmLBD* *11*	*Zm00001eb051620*	251	25,553.79	7.59	52.44	76.85	−0.037
*ZmLBD* *12*	*Zm00001eb052620*	211	21,657.7	9.13	47.7	76.49	0.004
*ZmLBD* *13*	*Zm00001eb060210*	164	17,625.74	7.61	73.43	63.9	−0.538
*ZmLBD* *14*	*Zm00001eb108240*	212	21,648.61	9.03	81.23	71.46	−0.096
*ZmLBD* *15*	*Zm00001eb117380*	683	74,595.77	4.52	52.19	71.92	−0.466
*ZmLBD* *16*	*Zm00001eb123060*	261	26,781.33	7.63	55.93	74.87	−0.011
*ZmLBD* *17*	*Zm00001eb125420*	298	32,356.28	7.66	50.71	72.75	−0.471
*ZmLBD* *18*	*Zm00001eb134030*	275	29,785.4	4.94	61.88	65.05	−0.388
*ZmLBD* *19*	*Zm00001eb145150*	260	26,721	7.64	41.33	71.08	−0.085
*ZmLBD20*	*Zm00001eb149360*	251	27,297.14	6.71	52.26	87.93	−0.091
*ZmLBD21*	*Zm00001eb151910*	221	22,980.32	8.1	55.62	80.05	0.038
*ZmLBD22*	*Zm00001eb167220*	413	43,899.25	5.38	49.32	64.84	−0.46
*ZmLBD23*	*Zm00001eb178740*	373	40,024.3	5.58	46.77	61.8	−0.461
*ZmLBD24*	*Zm00001eb191160*	216	22,426.46	8.1	45.27	78.52	0.095
*ZmLBD25*	*Zm00001eb218120*	199	20,689.5	7.65	54.94	73.67	−0.07
*ZmLBD26*	*Zm00001eb219100*	299	31,795.7	6.82	53.72	68.93	−0.368
*ZmLBD27*	*Zm00001eb265490*	221	23,002.21	6.03	52.15	88.64	0.18
*ZmLBD28*	*Zm00001eb269620*	229	24,347.37	7.6	84.18	62.36	−0.556
*ZmLBD29*	*Zm00001eb272570*	218	23,124.11	9.15	61.78	69.27	−0.348
*ZmLBD30*	*Zm00001eb286410*	195	20,845.92	5.9	46.23	80.15	0.005
*ZmLBD31*	*Zm00001eb288160*	278	28,814.73	8.5	28.84	72.66	0.003
*ZmLBD32*	*Zm00001eb292990*	206	21,875.46	8.53	71.27	66.55	−0.554
*ZmLBD33*	*Zm00001eb311030*	161	17,275.49	9.72	65.09	57.83	−0.546
*ZmLBD34*	*Zm00001eb326200*	215	22,175.3	8.85	72.25	70.42	−0.054
*ZmLBD35*	*Zm00001eb345960*	219	22,316.3	9.1	63.05	83.2	0.189
*ZmLBD36*	*Zm00001eb349310*	276	28,184.69	6.29	43.66	66.99	−0.035
*ZmLBD37*	*Zm00001eb353210*	283	29,602.54	5.57	50.58	87.39	−0.106
*ZmLBD38*	*Zm00001eb353230*	313	33,373.53	5.37	67.38	80.54	−0.354
*ZmLBD39*	*Zm00001eb355150*	212	22,149.29	7.06	54.3	85.42	0.086
*ZmLBD40*	*Zm00001eb365880*	270	27,773.26	8.52	42.82	67.04	−0.109
*ZmLBD41*	*Zm00001eb395110*	229	23,611.66	8.48	29.86	81.79	0.049
*ZmLBD42*	*Zm00001eb397130*	239	25,340.56	5.6	62.97	73.56	−0.227
*ZmLBD43*	*Zm00001eb398670*	265	27,061.63	8.26	69.94	73.7	−0.18
*ZmLBD44*	*Zm00001eb403030*	232	24,697.23	5.37	47.76	66.68	−0.271
*ZmLBD45*	*Zm00001eb405850*	490	54,339.82	4.99	56.15	90.31	−0.138

## Data Availability

The original contributions presented in this study are included in the article/Supplementary Materials. Further inquiries can be directed to the corresponding authors.

## Supplementary Materials

The following supporting information can be downloaded at: https://www.mdpi.com/article/10.3390/plants14162600/s1, Table S1. The Primer sequences of ZmLBD12 used for RT-qPCR; Table S2. Primer for cloning and identification of ZmLBD12; Table S3. Ka/Ks values of duplicate gene pairs in maize; Table S4. The motif composition of ZmLBD gene in maize.
